# Surgical management of lobar cerebral cavernous malformations in children: a single-center experience

**DOI:** 10.1007/s00381-024-06433-5

**Published:** 2024-05-07

**Authors:** Ryszard Sordyl, Lukasz Antkowiak, Marta Rogalska, Michael Schroter, Izabela Rosol, Marcin Ciekalski, Antonina Radolinska, Marek Mandera

**Affiliations:** 1 Department of Pediatric Neurosurgery, Medical University of Silesia in Katowice, Katowice, Poland; 2 Department of Otorhinolaryngology and Oncological Laryngology, Faculty of Medical Sciences in Zabrze, Medical University of Silesia in Katowice, Zabrze, Poland

**Keywords:** Cavernous hemangioma, Cavernoma, Vascular malformation, Pediatric, Seizures

## Abstract

**Purpose:**

We aimed to determine the surgical indications and postoperative outcomes among pediatric patients with lobar cerebral cavernous malformations (CCMs).

**Methods:**

We retrospectively reviewed pediatric patients operated on for lobar CCM between March 2010 and August 2021. Indications for surgery included (1) intracranial hemorrhage, (2) symptomatic superficially located lesion, and (3) asymptomatic CCM in non-eloquent area in case of strong parental preferences. Patients presenting with seizures were assessed using Engel Epilepsy Surgery Outcome Scale.

**Results:**

Twenty-one patients were included. The predominant symptoms were seizures (57.1%), headaches (33.3%), and focal neurological deficits (23.8%). Patients were qualified for surgery due to symptomatic intracranial hemorrhage (47.6%), drug-resistant epilepsy (28.6%), and focal neurological deficits (9.5%). Three patients (14.3%) were asymptomatic. A gross total resection of CCM with the surrounding hemosiderin rim was achieved in all patients. The mean follow-up was 52 months. No patient experienced surgery-related complications. In all individuals with a preoperative first episode of seizures or focal neurological deficits, the symptoms subsided. All six patients with drug-resistant epilepsy improved to Engel classes I (67%) and II (33%).

**Conclusion:**

Surgical removal of symptomatic lobar CCMs in properly selected candidates remains a safe option. Parental preferences may be considered a sole qualifying criterion for asymptomatic lobar CCM excision.

## Introduction

Cerebral cavernous malformations (CCMs), also known as cavernomas, are low-flow vascular lesions comprised of dilated sinusoidal thin-walled blood vessels without intervening brain parenchyma. The abnormal vessels forming CCMs lack a muscular layer and are lined with a single layer of endothelial cells without tight junctions, thus promoting bleeding from the malformation [1, 2]. The annual risk of hemorrhage is estimated at 3.3%, increasing to as much as 18.2% within 3 years for CCMs which have previously bled [3]. Generally, 25% of all CCM cases occur in the pediatric population [4]. Moreover, pediatric CCMs appear more aggressive due to the higher bleeding risk and faster growth than their adult counterparts, rendering a tailored approach for this age group [5]. Although CCMs typically present with seizures, headaches, or neurological deficits, up to 14% of CCMs in children are detected incidentally [5–7]. While asymptomatic lesions should generally not be treated since the risk of a surgical intervention exceeds the risk of bleeding, individuals with symptomatic CCMs are considered optimal surgical candidates [3]. The risk of hemorrhage and associated neurological deterioration differs depending on the CCM location in the central nervous system (CNS); similarly, the perioperative risk varies significantly. Most CCMs are located within the supratentorial lobar compartment, accounting for 80% of CNS CCMs. These lesions are associated with the lowest perioperative risk compared to CCMs in the basal ganglia or brainstem [8]. While the risk of surgical intervention has to be balanced against the neurological sequelae following symptomatic CCM bleeding, it seems crucial to separately address cavernomas occurring in diverse locations. However, the data on the surgical approach in pediatric CCMs remain relatively limited. Therefore, through our single-institutional experience, we aimed to determine the indications for surgical intervention and to evaluate postoperative outcomes among pediatric patients with lobar CCMs.

## Materials and methods

### Study population and data collection

This retrospective study encompassed pediatric patients (age at admission < 18 years old) operated on between March 2010 and August 2021 in the Department of Pediatric Neurosurgery, Medical University of Silesia in Katowice, with radiologically and pathologically confirmed intracranial supratentorial lobar CCM. Routinely, magnetic resonance imaging (MRI) and/or computed tomography (CT) scans on admission were performed in all patients. Data on patient demographics, clinical presentation, seizure-related history, CCM characteristics, details of surgical procedure, and postoperative outcomes were obtained from the clinical records. Patients with cavernomas located within deep brain structures and those with less than 6 months of follow-up were excluded from the study.

### Preoperative management and treatment strategy

Criteria for surgical treatment in our study included symptomatic CCMs, located superficially within the cerebral hemispheres, and those with an intracranial hemorrhage (ICH). Additionally, due to the strong parental preferences or an increase in CCM size, asymptomatic CCMs located in superficial, non-eloquent areas were qualified for surgical removal. Patients presenting with seizures (both recently developed and due to drug-resistant epilepsy) or with focal neurological deficits were considered symptomatic. Patients with a symptomatic intracranial hemorrhage on admission were qualified for early (< 14 days from bleeding) or delayed (≥ 14 days from bleeding) surgery depending on their clinical condition (signs of increased intracranial pressure, progressive neurological deficits). All individuals presenting with seizures underwent preoperative electroencephalography (EEG) examination in order to determine the association between CCM location and seizure origin. Surgery was aimed at the total resection of the lesion with the excision of the surrounding hemosiderin rim to the greatest achievable extent.

### Clinical evaluation

A modified Rankin Scale (mRS) was used to evaluate the patient’s degree of disability on admission and postoperatively. The mRS score between 0 and 2 points was considered a favorable treatment outcome, whereas patients with postoperative mRS scores between 3 and 6 points were categorized as having an unfavorable outcome. Additionally, children presenting with seizures were assessed using Engel Epilepsy Surgery Outcome Scale [9]. Postoperatively, individuals falling into Engel I and II classes represented an improvement over the initial clinical presentation. The follow-up clinical data were gathered through direct phone calls or analysis of outpatient clinic documentation.

## Results

A total of 21 patients (7 female and 14 male) with a mean age of 11 ± 5.27 years (ranging from 2 to 17 years) were included in our study. Three patients (14%) had multiple cavernomas (supra- and infratentorial). Since only the supratentorial CCM manifested clinically in each patient, the surgical excision was aimed at the sole removal of the symptomatic supratentorial CCM.

The most common presenting symptoms were seizures (57.1%), followed by headaches (33.3%) and focal neurological deficits (23.8%). On admission, three patients (14.3%) were completely asymptomatic. Baseline patient characteristics are presented in Table 1.

**Table 1:** Characteristics of included patients

**Feature**	**Value**
**Number of patients**	21
**Mean age (SD)**	11 (5.27)
**Sex – female,** ***n*** **(%)**	7 (33)
**Mean short-term follow-up duration in days (range)**	7 (4–8)
**Presenting symptoms**	
Seizures, *n* (%)	12 (57.1)
Headache, *n* (%)	7 (33.3)
Nausea/vomiting, *n* (%)	5 (23.8)
Focal neurological deficits, *n* (%)	5 (23.8)
Asymptomatic, *n* (%)	3 (14.3)
**Feature qualifying for surgery**	
Symptomatic hemorrhage, *n* (%)	10 (47.6)
Early surgery (< 14 days from hemorrhage), *n* (%)	5 (23.8)
Delayed surgery (≥14 days from hemorrhage), *n* (%)	5 (23.8)
Drug-resistant epilepsy, *n* (%)	6 (28.6)
Focal neurological deficits, *n* (%)	2 (9.5)
Parental preferences, *n* (%)	3 (14.3)
**Lesion location**	
Frontal lobe, *n* (%)	6 (28.6)
Parietal lobe, *n* (%)	6 (28.6)
Occipital lobe, *n* (%)	2 (9.5)
Temporal lobe, *n* (%)	7 (33.3)
**Complete resection confirmed on MRI scans,** ***n(%)***	21 (100)
**Engel Epilepsy Surgical Outcome Scale for patients with drug-resistant epilepsy**	
Engel class I, *n* (%)	4 (67)
Engel class II, *n* (%)	2 (33)
Engel class III, *n* (%)	0 (0)
Engel class IV, *n* (%)	0 (0)
**Modified Rankin Scale scores on admission**	
0, *n* (%)	3 (14.3)
1, *n* (%)	11 (52.4)
2, *n* (%)	4 (19)
3, *n* (%)	3 (14.3)
4, *n* (%)	0 (0)
5, *n* (%)	0 (0)
6, *n* (%)	0 (0)
**Mean follow-up duration in months (range)**	52 (7-151)
**Modified Rankin Scale scores at final follow-up**	
0, *n* (%)	12 (57.1)
1, *n* (%)	9 (42.9)
2, *n* (%)	0 (0)
3, *n* (%)	0 (0)
4, *n* (%)	0 (0)
5, *n* (%)	0 (0)
6, *n* (%)	0 (0)

*SD* standard deviation, *MRI* magnetic resonance imaging

Initial imaging studies of 10 patients (47.6%) revealed signs of recent intracranial bleeding. Patients were qualified for surgical CM removal due to symptomatic hemorrhage (47.6%), drug-resistant epilepsy (28.6%), and focal neurological deficits (9.5%).

Patients qualified due to CCM bleeding were operated on urgently (< 14 days, range: 4–13 days, *n* = 5) or with a delay (> 14 days, range 45–460 days, *n* = 5). The cause of early qualification was symptoms of intracranial hypertension in three patients and persistent epileptic seizures in the remaining two children. In cases of delayed treatment, the criteria for surgical treatment included epileptic seizure related to the lesion (*n* = 3), recurrent bleeding episode (*n* = 1), and persistent focal neurological deficit (facial nerve paresis, *n* = 1).

CCMs classified as asymptomatic were incidentally found in the temporal lobes of three patients. The reasons for brain imagining included minor head trauma (one case), persistent headaches (one patient), and routine follow-up MRI performed in a patient previously treated for subtemporal fossa rhabdomyosarcoma (RMS)—resection, chemotherapy, and radiotherapy. All three patients were qualified for CCM excision based on parental preferences. In the case of a patient diagnosed with headaches, all symptoms resolved within a week and did not recur in further observation. In the short-term follow-up, no patient operated on for asymptomatic CCM developed a new neurological deficit, while in the long-term evaluation, slight impairment of short-term memory and concentration was reported in the case of a child previously treated for the subtemporal RMS (this treatment included brain irradiation three years earlier).

**Fig. 1:**
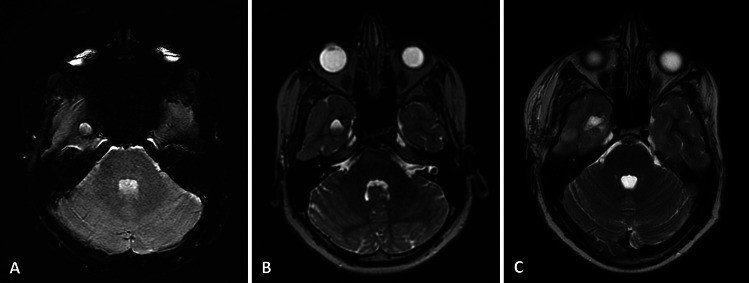
A 12-year-old male asymptomatic patient with an incidentally diagnosed CCM in the right temporal lobe. Preoperative MRI **A** SWI scan and **B** T2-weighted scan. The patient underwent gross total removal of the CCM. **C** Postoperative MRI T2-weighted scan confirmed gross total resection of the lesion

**Fig. 2:**
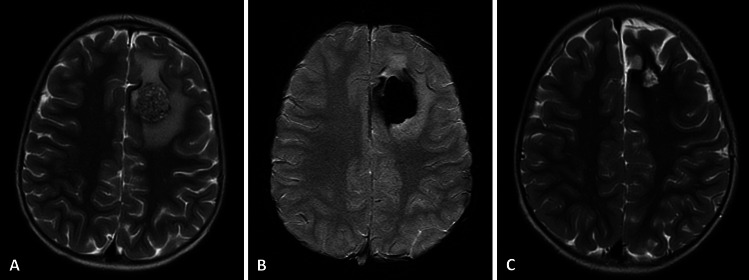
A 3-year-old male patient who presented with a first episode of seizures. Preoperative MRI **A** T2-weighted scan and **B** SWI scan showed CCM located in the left frontal lobe. The patient underwent gross total removal of the CCM. **C** Postoperative MRI T2-weighted scan confirmed gross total resection of the lesion

A gross total resection (GTR) of CCM with the surrounding hemosiderin rim was achieved in all patients and confirmed by a postoperative MRI scan (Figs. 1 and 2).

In the postoperative period, three individuals (14%) experienced transient complications. One patient developed a temporal subcutaneous fluid collection, one patient experienced a temporary swelling of his forehead and orbital region after a frontal craniotomy, while the remaining one (after the excision of CCM located in the occipital lobe) suffered from temporal vision impairment.

All patients were observed for the mean follow-up period of 52 months (ranging from 7 to 151 months). No patient experienced rebleeding. All individuals, who were primarily qualified for surgery due to the first episode of seizures, did not suffer from any seizure attacks postoperatively.

Among children with drug-resistant epilepsy, all patients (*n* = 6) experienced an improvement, with 4 patients (67%) falling into Engel class I and 2 patients (33%) categorized as Engel class II. All focal neurological deficits present on admission resolved in the long-term follow-up period.

**Fig. 3:**
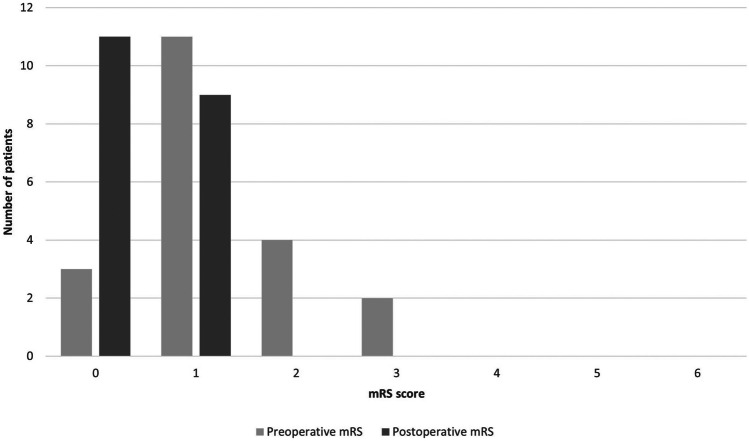
Comparison between pre- and postoperative modified Rankin Scale (mRS) scores

Overall, a favorable outcome based on the mRS score (0–2) was achieved in all patients. Only one patient (4.8%) that was preoperatively asymptomatic experienced an mRS score worsening after surgery (a child with a history of subtemporal RMS). Figure 3 illustrates the comparison between pre- and postoperative mRS scores.

## Discussion

It remains generally accepted that symptomatic supratentorial CCMs, especially those located in non-eloquent cortical areas, are suitable candidates for surgical excision. Considering longer life expectancy and higher accumulative risk of bleeding from CCMs in children than in adults, a more aggressive approach aimed at a GTR is warranted [4, 5, 10–12]. Following these qualifying criteria, the literature implies a remarkably high percentage of operative success (the lack of permanent postoperative complications)—up to 96% in some series—associated with a 99% rate of GTR [4, 7, 10]. In the present study, we achieved a 100% rate of GTR with the concurrent complete resolution of neurological symptoms in all preoperatively symptomatic patients. Nevertheless, even though all patients had a favorable outcome measured with mRS (0–2), in one of them, mRS increased compared to the preoperative state. However, the deterioration occurred in a child who had undergone cerebral irradiation about 3 years earlier due to RMS of the subtemporal fossa, which was probably the cause of later memory and concentration disorders. In fact, radiotherapy is a well-known risk factor for late-onset neurocognitive disorders (including memory loss) [13].

Additionally, our study included three patients with multiple CCMs (14%), which is consistent with epidemiological data suggesting the prevalence of hereditary CCMs accounting for 5–15% of all CCM cases [14].

While typically asymptomatic patients should be treated conservatively [5], the literature suggests that an increase in CCM size and strong parental preferences might shift the management approach towards an interventional one [5–7, 15]. Additionally, the permanent risk of progressive focal neurological deficits due to the bleeding episodes from conservatively managed CCM might constitute a psychological burden for the patient and their parents. Nevertheless, in such cases, the risk of postoperative neurological sequelae in a previously asymptomatic patient should be thoroughly discussed with the patient family. However, due to the greater neuroplasticity, pediatric patients are more likely to recover from iatrogenic neurological deficits than their adult counterparts.

Three children from our cohort were operated on despite being asymptomatic (one suffered from chronic headaches). They were qualified for surgery based on strong family preferences. No significant postoperative complications were found in those children. Moreover, the preoperative headaches reported in one case resolved entirely after surgery. Therefore, in our opinion, the presence of psychological burden in children’s caregivers may be considered a qualifying criterion for surgery in carefully selected individuals since the perioperative risk does not seem to significantly exceed the natural course of asymptomatic supratentorial CCMs that are located in non-eloquent brain areas.

Similar to adults, the most frequent initial clinical presentation of lobar CCMs in children remains seizures [2, 5, 10]. In our cohort, seizures on admission were present in 57.1% of patients, constituting the most common presenting symptom. Furthermore, we successfully attempted to remove the hemosiderin rim in all patients and observed complete postoperative resolution of seizures in all six patients whose first episode was a symptom of CCM bleeding. Moreover, all six patients with preoperative drug-resistant epilepsy also improved, categorizing them into Engel classes I (67%) and II (33%). Nevertheless, the impact of the hemosiderin rim removal on the withdrawal of seizures remains controversial [2, 5, 10, 16, 17]. In their multicenter cohort, Hugelshofer et al. reported a 100% rate of hemosiderin rim removal with concurrent 83% of patients pertaining to Engel classes I and II (72% and 11%, respectively) [16]. Contrarily, although in the study performed by Sawarkar et al., merely 41% of patients underwent complete hemosiderin rim removal, 100% of the study cohort fell into Engel classes I and II postoperatively (94.1% and 5.9%, respectively) [11]. Therefore, the discrepancies in the rate of postoperatively seizure-free patients might result from factors other than solely the extent of hemosiderin rim resection. It has been suggested that the coexistence of several epileptogenic factors (the cortical site, the frequent presence of calcifications in brain parenchyma surrounding lobar CCMs, and the iron-containing deposits in the direct vicinity of CCMs, as mentioned above) might contribute to the seizure attacks [10, 17].

### Limitations

Our study limitations include its retrospective character and small patient sample from a single center. Additionally, our follow-up clinical data were partially gathered by means of direct phone calls without the ability to examine patients during in-person follow-up visits.

## Conclusions

Surgical excision of symptomatic lobar CCM remains a safe and effective approach when based on the proper patient selection. Parental preferences may be considered the sole qualifying criterion for the operative removal of asymptomatic lobar CCMs. Complete symptom resolution in patients with the preoperative first seizure episode and improvement in drug-resistant epilepsy control was achieved with concurrent lesionectomy and complete hemosiderin rim removal. Nevertheless, further large prospective studies are highly warranted in order to precisely determine the role of hemosiderin rim excision on the postoperative withdrawal of seizures.

## Data Availability

The data generated during this study are available within the article. Datasets analyzed during the current study preparation are available from the corresponding author upon reasonable request.
